# Nonopioid Pain Protocols vs. Opioid Analgesia for Postoperative Pain Control Following Arthroscopic Surgeries: A Literature Review

**DOI:** 10.7759/cureus.83357

**Published:** 2025-05-02

**Authors:** Saif L Juma, Justin Ma, Janae L Rasmussen, Zachary Shorts, Marco Magardichian, Panayiotis Gentis, Bailey Patrick, Jonathan Pettegrew

**Affiliations:** 1 Orthopedic Surgery, Michigan State University College of Osteopathic Medicine, East Lansing, USA; 2 Orthopedic Surgery, California Health Sciences University College of Osteopathic Medicine, Clovis, USA; 3 Orthopedic Surgery, Valley Consortium for Medical Education, Modesto, USA; 4 Orthopedic Surgery, A.T. Still University School of Osteopathic Medicine, Mesa, USA; 5 Orthopedic Surgery, Ohio University Heritage College of Osteopathic Medicine, Dublin, USA; 6 Dermatology, University of Missouri, Columbia, USA; 7 Sports Medicine, Valley Orthopaedic Bone and Joint, Modesto, USA

**Keywords:** analgesia, arthroscopy, opioid dependence, opioids, orthopedic surgery, postoperative

## Abstract

Arthroscopic surgery is a widely used technique in orthopedic practice for treating various joint pathologies, including anterior cruciate ligament (ACL) tears, meniscal injuries, and rotator cuff tears. Arthroscopy offers a minimally invasive alternative to traditional surgical options, allowing for improved recovery times and reduced soft tissue damage. Despite its minimally invasive nature, postoperative pain management remains a critical component of recovery. Traditionally, opioids have been the cornerstone of analgesia following arthroscopy. However, rising opioid overdose rates and the growing awareness of opioid dependence have brought increased attention to the role of orthopedic surgeons, who are among the highest prescribers of opioids. This has led to a broader exploration of nonopioid and multimodal analgesic strategies aimed at reducing opioid consumption. Optimal pain control following arthroscopy must balance efficacy and safety through individualized strategies that account for variability in patient demographics, surgical procedures, preoperative opioid use, and social determinants of health. Nonopioid agents, such as nonsteroidal anti-inflammatory drugs (NSAIDs), acetaminophen, gabapentinoids, and local anesthetics, have been examined for their varied mechanisms of pain relief and potential to reduce opioid dependence. Despite promising outcomes with these agents and the introduction of multimodal analgesia protocols, postoperative prescribing practices remain inconsistent across arthroscopic surgeries. This literature review compares opioid-based and nonopioid pain management strategies following arthroscopic surgery, evaluating their effectiveness in pain control, patient satisfaction, and complication rates. It also examines the risks associated with prolonged opioid use and emphasizes the importance of individualized pain management that considers both clinical and social factors. Recent literature analyzing multimodal analgesic regimens following arthroscopic surgery highlights which protocols yield better outcomes. Multimodal nonopioid protocols were found to provide comparable or improved pain control while significantly reducing opioid consumption and associated side effects. NSAIDs, acetaminophen, gabapentinoids, regional anesthetics, and intra-articular opioid administration demonstrate efficacy in optimizing postoperative pain control while minimizing opioid reliance. Additionally, multimodal regimens are associated with better functional outcomes, lower complication rates, such as constipation and nausea, and a reduced risk of prolonged opioid dependence. However, inconsistency in analgesic protocols and variability in patient factors continue to challenge standardization. Further high-quality research is necessary to establish consistent, evidence-based postoperative analgesia guidelines for arthroscopic surgery, with an emphasis on minimizing opioid overprescription and enhancing patient outcomes.

## Introduction and background

Arthroscopy, a common orthopedic procedure, revolutionized orthopedic surgery by offering a minimally invasive option compared to other surgical approaches [1]. Arthroscopy allows for visualization of a joint with a camera and minimally invasive incisions. Many orthopedic pathologies, such as anterior cruciate ligament (ACL) tears, meniscus tears, and rotator cuff tears, are treated using arthroscopy as the standard of care. Like other orthopedic surgeries, it requires effective postoperative pain management. Opioids have been used traditionally to control postoperative pain in orthopedic surgery. However, drug overdose rates have steadily increased each year, with opioids responsible for nearly two-thirds of these cases, making them the leading cause of accidental death in the United States [2,3]. Prescriptions for opioids increase the risk of dependence, especially with long-term utilization [3].

Analgesics are essential for minimizing postoperative pain following arthroscopic surgeries. They can be divided into two main groups: opioids and nonopioids. With opioid-induced analgesia, the mechanism of action primarily focuses on activating mu-opioid (MOP) receptors in the midbrain, which stimulates descending inhibitory pathways in the central nervous system, and suppresses nociceptive transmission from the periphery to the thalamus [4]. This mechanism makes opioids particularly effective for postoperative pain management due to stimulating the MOP. In comparison, the most widely used nonopioid analgesic agents, such as nonsteroidal anti-inflammatory drugs (NSAIDs), local anesthetics, and typical antidepressants, have similar physiological effects as opioids. However, they greatly differ in their pharmacological mechanism. Nonopioid analgesics alleviate pain by a range of mechanisms. NSAIDs inhibit cyclooxygenase (COX) enzymes to reduce inflammation [5]. Many local anesthetics block voltage-gated sodium channels on the internal surface of nerve cell membranes, which decreases signaling [5]. Antiepileptic medications reduce calcium-dependent release of excitatory neurotransmitters, making them effective in minimizing peripheral and central pain [5]. The various mechanisms and pathways of nonopioid analgesics provide a variety for pain management optimization. Understanding the contrasting mechanisms of both opioid and nonopioid agents allows for the potential to improve modern analgesia effectiveness and improve recovery outcomes following arthroscopic surgeries.

Orthopedic surgeons are among the highest prescribers of opioids and, in certain instances, exceed opioid postoperative prescribing guidelines [6,7]. The American Academy of Orthopaedic Surgeons (AAOS) currently recommends evidence-based prescribing practices and standardized opioid protocols for orthopedic procedures [8]. The opioid epidemic has compelled increased investigation into nonopioid pain management modalities for arthroscopic surgical patients [9,10]. Although extensive research has investigated opioid use following procedures such as total joint arthroplasty, there is limited literature comparing nonopioid pain protocols to opioid analgesia for postoperative pain control after arthroscopic surgeries [11]. Perioperative prescribing habits have significant long-term consequences, since some opioid-naive patients who have orthopedic surgery can become opioid dependent [9]. As orthopedic surgeons are among the top prescribers of opioids, they play a key role in addressing the ongoing opioid crisis by refining pain management strategies [11].

This review evaluates the effectiveness of postoperative pain management protocols following various arthroscopic procedures. It evaluates the application of opioid analgesia in arthroscopic surgery, particularly with patient clinical outcomes. It also evaluates the effectiveness of nonopioid pain protocols, including NSAIDs, acetaminophen, gabapentinoids, and regional anesthesia following arthroscopic procedures. This review highlights the importance of multimodal analgesia in optimizing pain control to reduce the incidence of opioid dependence while improving postoperative pain control.

Methods

A comprehensive literature search was conducted by six authors (SJ, JM, ZS, MM, PG, BP) to identify studies published between 2000 and 2025, utilizing databases such as PubMed and Google Scholar. Keywords were used in various combinations, including “arthroscopic surgery,” “postoperative opioid use,” “opioid analgesia,” “nonopioid pain management,” “NSAIDs,” and “multimodal analgesia.” Only studies evaluating postoperative pain control following arthroscopic procedures, including knee, shoulder, and hip surgeries, were included. Studies were considered if they compared opioid-based analgesia with nonopioid pain protocols and assessed functional recovery with patient outcomes. Studies were excluded if they were published before 2000, focused on invasive orthopedic procedures rather than arthroscopic techniques, or did not specifically examine postoperative pain management strategies. No meta-analysis was conducted. This review is based on a literature search and is not a formal systematic review; therefore, some relevant studies may not have been included due to the search parameters.

## Review

Risk of opioid dependence

Postoperative opioid dependence is a risk factor for opioid use. Opioid dependence is not solely based on the individual behaviors of the patient, as it is influenced by social determinants of health (SDOH) and health literacy. A cross-sectional study highlights how the social vulnerability index (SVI) was strongly correlated with higher rates of drug overdoses and misuse [12]. The score comprises many SDOH, such as socioeconomic status, disability, minority status, and access to transportation. Physicians should consider these factors when prescribing opioids. Interventions such as strengthening social support systems, increasing economic stability, and promoting education and health literacy are paramount. A study of 445 adults living with chronic pain examined the health literacy of patients and the relationship to opioid misuse [13]. The fcy couldindings showed a negative correlation between health literacy and opioid misuse [13]. This suggests that enhancing health literacy plays a crucial role in reducing opioid abuse and pain management. Individuals in underserved communities often face barriers to quality healthcare [12]. However, there are higher rates of overdose in counties with a higher percentage of primary care physicians [12]. Several factors may contribute to higher rates of opioid dependence and overdose, including opioid prescription rates, clinical practices, and the effectiveness of public health policies. Further investigation is warranted to understand this association to help physicians better care for their patients by considering social and health literacy factors.

Furthermore, an analysis of 2,214 counties in the US in 2016 put together an extensive list of SDOH characteristics that were most prevalent in 8.7% of these counties that were considered to have high population rates of opioid-related inpatient stays and emergency department visits [14]. Patients from urban, densely populated, and more racially segregated communities, as well as patients who were located in areas with greater pharmacy density, higher population of Medicaid enrollment, high crime rates, and higher percentage of economically disadvantaged residents, were more likely to have these visits [14]. Not only did location play a role, but patients from single-parent households and less religious affiliation were also more likely [14]. This study shows that many factors, even geographical location, can influence patients' risk of opioid dependence. Physicians, especially those situated in these locations, must be aware of these factors. Another study analyzed the demographics that were most prone to opioid use disorder (OUD) in the perioperative and long-term setting [15]. Hispanic populations have the largest proportion of youth with OUD, and this is often attributed to a lack of translation services available, while caucasian patients receive more pain management, but are at higher risk of developing OUD [15]. African-American patients suffer from the criminalization aspect of OUD, and Native American patients often receive care in low-funded communities and thus lack the resources to combat OUD [15]. Lastly, especially vulnerable populations, like those without housing or formerly incarcerated, often lack access to healthcare and insurance, with a higher risk of opioid overdose [15]. Physicians, especially orthopedic surgeons, must be aware of these demographic disparities and outcomes by race to understand how they can reduce the risk of their patients becoming part of the opioid epidemic.

Opioid analgesia in arthroscopic surgery

Orthopedic surgeons should consider many factors when prescribing opioids following arthroscopic surgery, such as adverse effects, effectiveness in controlling pain, dosage and length of prescription, patient characteristics and risk profiles, and potential abuse following the intended prescription. There are several benefits to postoperative opioid use for immediate postoperative pain control, but opioid use also carries the risk of dependence. A randomized controlled trial found that prescribing 30 opioid tablets, instead of 60, after hip arthroscopy significantly reduced the number of remaining tablets without compromising postoperative pain control [16]. Additionally, preoperative factors, such as prior opioid use and international hip outcome tool scores (iHOT-12), were predictive of postoperative opioid requirements [16]. The iHOT-12 uses 12 questions from the original iHOT-33, designed to measure the impact of hip disease in active patients, as well as the effect of treating the disease presented [17]. This patient-reported measure of quality of life can be given preoperatively and/or postoperatively, with scores calculated as an average ranging from 0 to 100, with 100 indicating the best quality of life [17]. This study demonstrates that there is no additional benefit in prescribing significantly more opioid tablets postoperatively to manage pain following hip arthroscopy [16]. They also found that preoperative iHOT-12 scores heavily predicted postoperative opioid consumption [16]. Evidence demonstrates the importance of conducting a patient history and considering factors such as SDOH to help orthopedic surgeons predict the appropriate postoperative opioid requirements.

The increased utilization of opioids postoperatively has been seen in other arthroscopic surgeries besides hip arthroscopy. A randomized controlled trial that analyzed 71 patients who underwent arthroscopic meniscal surgery or anterior cruciate ligament reconstruction (ACLR) measured median post-discharge consumption of prescribed oxycodone [18]. All meniscus patients and 71% of ACLR patients stopped opioid consumption within one week after surgery, and almost one-third (32%) of patients following either surgery (the majority being meniscus surgeries) took no opioids after surgery [18]. This led to the suggestion that reasonable prescription quantities are 20 tablets of 5 mg oxycodone after ACLR or five tablets of 5 mg oxycodone after arthroscopic meniscus surgery [18]. This indicates that the quantity of opioids prescribed postoperatively could be tailored based on the specific type of arthroscopic procedure. Additionally, as demonstrated in both this study and the hip arthroscopy study discussed previously, shorter prescription durations can still effectively manage postoperative pain. Decreasing the amount of prescribed opioids can impact patient expectations of what is appropriate to use postoperatively, resulting in less opioid utilization.

Similarly, a nonrandomized controlled trial was conducted with 20 patients who had undergone ACLR (10) or bridge-enhanced ACLR (10) [19]. Across both groups, oxycodone was oversupplied on average by 46 pills per patient, which correlates to a greater than 70% unused opiate rate, calculated as the amount of opioids not taken as a percentage of that which was prescribed [19]. This reinforces the evidence that orthopedic surgeons are overprescribing opioids, which contributes to the increased incidence of opioid dependence. Overprescribing opioids increases the risk of dependence and abuse, as seen in a randomized controlled trial that followed 176 patients who underwent a variety of different arthroscopic surgeries [20]. Orthopedic surgeons prescribed a mean of 26.6 opioid pills, but patients reported taking an average of 15.5 pills [20]. The mean morphine milligram equivalent (MME) consumption varied across the different procedures, with a total of 39% of the total opioid prescription being utilized across all five procedures (5 mg hydrocodone-324 mg acetaminophen or 5 mg oxycodone-325 mg acetaminophen were given based on surgeon preference) [20]. Additionally, 7% of patients continued opioid use at three weeks postoperatively, and 24% of the patients with remaining medication intended to keep their medication for use in the future [20]. This study is consistent with others, highlighting that orthopedic surgeons, in general, overprescribe opioids for arthroscopic surgeries. Overprescribing may lead to patients saving remaining opioids for use in the future, which increases the likelihood of opioid abuse.

Moreover, certain factors can increase or decrease the likelihood of persistent opioid abuse following arthroscopic surgeries. In a retrospective cohort study, persistent opioid use (two or more prescriptions filled between nine and 15 months post-op) was measured between opioid-naive (no opioid prescriptions filled within one year prior to the surgery) and non-naive populations following hip arthroscopy [21]. Of these patients, 1,525 were considered opioid-naive, and 384 were non-naive [21]. They found that preoperative opioid use (47% of non-naive patients versus only 2% of opioid-naive patients), as well as older age, were associated with increased risk of persistent postoperative opioid use [21]. Another prospective cohort study found that patients with a self-harm disorder, mood disorder, female gender, anxiety disorder, alcohol abuse, history of pain diagnosis, or patients who filled an opioid prescription in the 30 days prior to surgery, had an increased chance of prolonged opioid abuse [22]. These studies demonstrate that those who have experience with prior opioid use, as well as other factors such as prior mental health diagnosis, are more likely to use opioids inappropriately, such as when pain is controlled, following arthroscopic surgery.

Opioid use postoperatively after arthroscopy has some potential benefits. A quantitative meta-analysis on the efficacy and side effects of intra-articular morphine in patients who underwent knee arthroscopy was compared to a control saline placebo group [23]. This study found that a single dose of intra-articular morphine at the end of arthroscopic knee surgery reduced the need for supplementary pain relief, increased the time interval before the first request for additional analgesics was needed, and provided overall better pain relief, all with short-term side effects (nausea, vomiting, urinary retention, pruritus, tiredness, headache) similar to the saline placebo [23]. Pain scores were typically assessed during the early postoperative period, with most studies evaluating patient-reported outcomes within the first 7-14 days following surgery. This study highlights that the route of opioids can significantly affect postoperative pain, such as intra-articular injection compared to oral administration. Due to variability in opioid route and type, MME calculations are used to help determine a patient’s cumulative intake of any opioids to help reduce too high of doses. A prospective multicenter observational study gathered 100 patients who underwent knee arthroscopy, and patients were prescribed a median of five opioid pills consisting of 37.5 oral MME [24]. All patients consumed less than or equal to five pills, and 92% of patients discontinued opioids two days after surgery [24]. Despite this limited prescription capacity, all patients were highly satisfied with their pain coverage and asked for no refills [24]. This recent literature demonstrates that opioids are a potent and effective method of efficient pain control, especially if utilized intraarticularly for arthroscopic surgeries. Additionally, oral opioids can be safely utilized by patients with appropriate pain and opioid use expectations and low risk for opioid dependence.

Nonopioid pain protocols for arthroscopic surgery

Given the significant side effects associated with opioid analgesics, recent research has increasingly explored the effectiveness of nonopioid pain protocols, including NSAIDs, acetaminophen, and regional anesthesia for managing postoperative pain following arthroscopic surgery. A randomized, double-blind, placebo-controlled trial was conducted demonstrating nonopioid (NSAIDs such as naproxen and ibuprofen) usage in comparison to a placebo after arthroscopic rotator cuff repair [25]. Both sample sizes were provided with MME, with the NSAID group provided with 168 MMEs relative to the placebo group's 211 MMEs. The trial monitored postoperative effects, impact on tendon healing, and potential side effects of usage [25]. Using the visual analog scale (VAS) pain scores within the first postoperative week, the NSAID group demonstrated lower pain scores compared to the placebo group. Specifically, mean VAS scores were significantly lower in the ibuprofen group on postoperative days three (2.6 vs. 3.6), four (2.3 vs. 3.1), five (1.9 vs. 2.8), and six (1.8 vs. 2.7), with all differences reaching statistical significance (p ≤ 0.04) [25]. The NSAID group also showcased higher American Shoulder and Elbow Surgeons Shoulder Scores (ASES) and greater shoulder forward flexion (162° vs. 153° at six months), indicating improved postoperative function compared to the placebo group [25]. The implications of the study suggest that NSAIDs could potentially lower the amount of opioid prescriptions. Additionally, NSAIDs can help contribute to improved patient-reported outcomes, indicated by improved ASES/VAS scores.

Similar conclusions were indicated in the study done by Gazendam et al. [26] on the usage of nonopioids for pain control following knee and shoulder arthroscopic surgeries. A multicenter randomized controlled trial was conducted with two groups: nonopioids (naproxen 500 mg twice per day (BID) as needed (PRN) and acetaminophen 1,000 mg every six hours (Q6H) PRN, a limited rescue opioid prescription of hydromorphone 1 mg Q4H PRN), and standard care with opioids based on the surgeon's practice preferences [26]. Patients were evaluated two weeks and six weeks postoperatively on treatment satisfaction, pain rating using VAS, and oral morphine equivalents (OMEs) prescribed and refilled [26]. Results of the study indicated the nonopioid group consumed fewer OMEs (25 OMEs ± 10) relative to the standard care group (100 OMEs ± 40) [26]. Pain scores based on the VAS were compared, and there was no significant difference (p > 0.05) in reported pain levels [26]. Patient satisfaction measured via the Hospital Consumer Assessment of Healthcare Providers and Systems (HCAHPS) questionnaire indicated similar satisfaction levels between groups [26]. The results of Gazendam et al. [26] indicate that nonopioid usage reduces opioid usage without compromising pain control, provides adequate pain relief, and maintains patient satisfaction. Both studies showcase how NSAIDs would be beneficial alternatives for opioid usage reduction.

Local anesthetics, such as bupivacaine, have also been introduced as an alternative to improve pain relief and reduce opioid usage. Many orthopedic surgeons elect to have preoperative nerve blocks placed to help with both intraoperative and postoperative pain control, with the average response time of benefit being 12-18 hours [27]. Bupivacaine acts as a sodium channel blocker, inhibiting the influx of sodium ions into the neuron membrane [28]. This action prevents the needed voltage to cause depolarization, preventing the initiation or conduction of a pain signal [28]. Ekelund et al. conducted a study on bupivacaine, its effect on pain relief, and lower usage of opioids post-arthroscopic shoulder subacromial decompression [29]. The study was a randomized, placebo-controlled trial, which measured pain scores, opioid consumption, and patient satisfaction between the experimental group [29]. The experimental group used a SABER-bupivacaine local anesthetic injection, while the placebo group received saline in the subacromial space [29]. The outcome was a mean of a significant 1.3-point reduction on a 0-10 pain scale with the experimental group compared to the placebo group for postoperative range-of-movement pain over 72 hours [29]. The experimental group also took a mean time of 12.4 hours compared to 1.2 hours for the placebo group to request opioid rescue medication [29]. This study highlighted how local anesthetics can help reduce postoperative pain, reducing the dosage needed for pain relief with opioids [29]. Nonopioids such as NSAIDs, acetaminophen, and local anesthetics have demonstrated the potential to reduce reliance on opioid usage and provide valuable alternative treatments for postoperative pain relief following arthroscopic surgical procedures.

Comparison of opioid vs. nonopioid pain management strategies

With regard to modern postoperative pain management, particularly arthroscopy, emerging research challenges the necessity of high-dose opioid prescriptions. Following shoulder arthroscopy, Kishan et al. reported that dispensing fewer opioid pills results in equal or more effective pain management [30]. Supplying a decreased quantity of opioids for postoperative pain management can minimize potential side effects, as well as medication misuse, leading to an overall improved outcome. In addition, an observational study with a sample of 100 people found that 90% of patients consume five or fewer opioid pills following knee arthroscopy, with over half of the patients consuming zero pills [24]. This highlights that most patients are able to recover comfortably without or with very few opioids. Alternatives, such as NSAIDs or acetaminophen, are often sufficient without opioids. With arthroscopy’s minimally invasive nature, there is typically less postoperative pain compared to a more invasive procedure for the same pathology [31]. In a cross-sectional comparison of pain management in post-arthroscopic surgery, when given nonopioid pain medications, patients on average took them for 9-10 days for pain management, in contrast to opioids, which averaged seven days [32]. In accordance with the data presented, nonopioid regimens can provide adequate pain control following arthroscopy, while in turn, reducing the risks associated with opioid use. More evidence demonstrates that opioids may not be necessary for postoperative pain, but there are limitations, especially considering the type of arthroscopic surgery and patient expectations.

The management of postoperative pain following arthroscopic surgery has various side effects and risks associated with its respective treatments. Even though commonly prescribed, opioid agents can have adverse effects, notably the risk of dependence, leading to the consideration of alternative therapies. Stephan et al. demonstrated that postoperative opioid use for pain management hinders the body’s natural production of beta-endorphins and suppresses the expression of mu-opioid receptors, causing an increase in overall pain intensity and duration following surgeries [33]. The use of opioids is increasingly being approached with caution as a result of opioids interfering with endogenous analgesics, ultimately causing longer-lasting pain. Opioids compared with nonopioids, for post-arthroscopic analgesia, patients who used opioids experienced significantly more days with gastrointestinal issues, including constipation and gastrointestinal discomfort [34]. The adverse effects of opioids highlight the benefit of alternative treatments and multimodal analgesia. The typical postoperative analgesic agents, such as NSAIDs, reduce other opioid-induced adverse effects, such as nausea, vomiting, and sedation postoperatively [35]. However, they significantly impair renal function, causing potential acute kidney injury or, with prolonged use, chronic renal failure [35]. Therefore, NSAIDs are typically used with caution in patients with a history of renal disease. Additionally, NSAIDs are under investigation for their potential bone repair process and delayed healing or non-union of osseous tissue [36]. The inhibition of COX enzymes via NSAIDs reduces the synthesis of prostaglandins, causing a risk of reduced functionality in endogenous bone remodeling [36]. There remains debate within the orthopedic community regarding the use of NSAIDs for osseous healing considerations. Given the limitations associated with both opioid and nonopioid analgesics, it is essential to further explore multimodal pain management strategies that balance efficacy with safety.

Recovery and rehabilitation are equally important when it comes to treatment considerations for postoperative pain management. Multiple variables are taken into account, including adequate pain control, patient expectations, the extent of anticipated regained functionality, and favorable rehabilitation outcomes. Long-term opioid usage has been shown to result in reduced functionality and additional opioid prescriptions [34,36]. Although opioids are reliable in pain management, their therapeutic benefits do not extend beyond short-term analgesia. Instead, their prolonged use is detrimental to physical recovery. Conversely, evidence suggests nonopioid agents and regimens were found to have comparable pain relief to opioids but also allowed earlier return to physical activity, resulting in improved patient-reported outcomes [37]. Taking into account each of these factors, nonopioid analgesics demonstrate great promise in comprehensive outcomes. Moreover, the type of analgesia used for treatment has an overall effect on each patient's quality of life. As a result, when evaluating postoperative pain management strategies, it is important to have preoperative discussions with patients regarding their medical and social history and expectations for pain control and recovery.

Role of multimodal analgesia in reducing opioid use

Multimodal analgesic regimens have become expected practice standards to control pain following arthroscopic surgery. These regimens employ a combination of drugs acting on multiple pain pathways to achieve maximal analgesia [2]. The American Society of Anesthesiologists (ASA) recommends routine use of these regimens where feasible [38]. The principal aim of these regimens is to reduce postoperative opioid consumption, which reduces the risk of dependence.

Recent literature has demonstrated that multimodal analgesia effectively enhances pain management while reducing reliance on opioids [3,34,37,39]. These studies used the VAS to measure postoperative pain in patients following common arthroscopic procedures [3]. All patients received a standardized nonopioid multimodal analgesic regimen that included preoperative oral drugs (celecoxib 400 mg, acetaminophen 975 mg, gabapentin 300 mg, tramadol 50 mg) and intravenous dexamethasone (8 mg) [3]. Intraoperative local infiltration analgesia (ropivacaine 150 mg, ketorolac 30 mg, epinephrine 1 mg) was administered along the surgical incision, intraoperatively [3]. In the multimodal protocol, celecoxib - a selective COX-2 inhibitor - was used preoperatively to provide anti-inflammatory and analgesic effects while reducing gastrointestinal side effects compared to nonselective NSAIDs, an important consideration when multiple medications are combined. Postoperatively, patients were on a structured pain control regimen for two weeks, followed by acetaminophen (1,000 mg every eight hours as needed) from postoperative days 15-28 [3]. Pregabalin (75 mg) was also provided as an alternative to gabapentin in intolerant patients [3]. NSAIDs were used for inflammation, gabapentin was used for neuropathic pain, diazepam was utilized to alleviate muscle cramps, and frequent icing was recommended [3,34,38,40]. Overall, this protocol combines anti-inflammatories, neuropathic agents, muscle relaxants, and local anesthetics to optimize pain control and reduce opioid use after arthroscopic surgery.

This multimodal nonopioid protocol was initially implemented in a cohort study by Moutzouros et al. following standard arthroscopic procedures, such as rotator cuff repair, ACLR, partial meniscectomy, and shoulder labrum repair [3]. The regimen employed preoperative analgesics, intraoperative local infiltration, and the described postoperative regimen [3]. The population consisted of 141 patients, 45% of whom were not given postoperative opioids and had adequate pain control [3]. The remaining 55% needed minimal rescue opioids, which were primarily based on pain severity, nature of surgical procedure, and mental health history [3]. A Spearman correlation showed that higher oxycodone consumption correlated with increased pain at the first postoperative visit (r = 0.40, p < 0.001) [3]. These findings suggest that the individualized multimodal nonopioid pain protocol can be an effective alternative to traditional opioid analgesia.

In another study, Jildeh et al. investigated the effectiveness of multimodal analgesia following arthroscopic rotator cuff repair [34]. Forty patients were enrolled and randomly assigned to two treatment groups: 23 patients were administered routine opioid-based pain relief, and 17 patients were administered a nonopioid regimen [34]. The opioid-free regimen was associated with decreased pain scores (VAS and patient-reported outcome measurement) at all measured intervals (p < 0.01) [34]. Additionally, the patients treated with opioids showed significantly higher rates of constipation (p = 0.003) and nausea (p = 0.020) [34]. These findings indicate that multimodal nonopioid therapy can offer pain relief equal to or even greater than traditional opioid analgesia, but with fewer side effects. In another randomized controlled trial by Jildeh et al., 61 meniscus surgery patients were randomly assigned to opioid (n = 30) and nonopioid (n = 31) analgesia groups [40]. Outcome measures showed no group differences in VAS scores for pain after controlling for confounding variables [40]. The study upheld that a multimodal nonopioid regimen provided comparable pain relief, patient outcomes, and side effects to traditional opioid analgesia after primary meniscus surgery. Jildeh et al. also evaluated a multimodal nonopioid regimen after arthroscopic shoulder labral surgery in 48 patients randomized into equal opioid and nonopioid groups [38]. No differences in mean VAS scores were noted within the first 10 postoperative days (p > 0.05) [38]. The study concluded that multimodal non opioid analgesia was equally effective as traditional opioid analgesia regarding pain control, side effects, and patient satisfaction. Through its action on multiple pain pathways at one time, multimodal analgesia decreases each drug's dosage requirement, thus diminishing side effects, opioid dependency, and improving patient outcomes.

A summary of the studies included in this review is provided in Table 1.

**Table 1:** Summary of reviewed studies evaluating nonopioid vs. opioid analgesia following arthroscopic surgery VAS = visual analog scale, NSAIDs = nonsteroidal anti inflammatories, iHOT-12 = International Hip Outcome Tool, ACLR = anterior cruciate ligament reconstruction, KOOS = knee injury and Osteoarthritis Outcome Scores, MME = morphine milligram equivalents, OMEs = oral morphine equivalents, ASES = American Shoulder and Elbow Surgeons, SST = simple shoulder test, PROMIS-PI = Patient-reported Outcome Measurement Information System, QoR-9 = Quality of Recovery survey 9, APS-POQ-R = Revised American Pain Society Patient Outcome Questionnaire

Author(s) & year	Variables Evaluated	Outcomes	Clinical importance
Moutzouros et al., 2020 [3]	Multimodal nonopioid pain protocols, if rescue opioids were needed, and patient pain reporting using the VAS pain score.	A prospective study found that 45% of patients did not need any breakthrough prescription opioids. Patients who did require rescue opioids were more likely to have a history of anxiety/depression.	Sufficient pain management can be achieved without using opioids in certain patient populations. Multimodal pain protocols have a promising future in common orthopedic sports procedures.
Hartwell et al., 2020 [8]	Total postoperative opioid consumption based on optional opioid prescription or multimodal pain management with opioids included.	A randomized controlled trial found that there was no significant reduction in the number of opioid tablets used between groups. There was a reduction in the amount of unused tablets by the group that was not initially given opioids, but the option to fill the prescription if needed.	Offering optional opioid prescriptions compared to including them in the multimodal pain management plan can reduce the amount of unused opioid tablets.
Gazendam et al., 2022 [9]	Postoperative opioid consumption when given an opioid analgesic versus an opioid-sparing pain management protocol.	A randomized control trial was completed on 193 patients, which found that multimodal opioid-sparing postoperative pain management protocols significantly reduced postoperative opioid consumption over six weeks, compared to standard pain management protocols. Fewer medication-related adverse effects were reported in the opioid sparing protocol.	Opioid consumption can be significantly reduced when a multimodal opioid-sparing pain management protocol is implemented. This can also decrease the amount of adverse effects caused by opioids.
Kamdar et al., 2020 [10]	Total postoperative opioid consumption, preoperative opioid consumption (defined as within three months of surgery), smoking status, and history of depression.	A retrospective study asked patients about their opioid use around the time of knee arthroscopy. Of the 100 patients, 90% consumed five or fewer opioid pills (oxycodone 5 mg) following knee arthroscopy, with more than half of the patients consuming zero pills.	Five or fewer 5 mg oxycodone tablets were sufficient for the majority of patients’ pain control in a retrospective cohort of 100 patients who had undergone knee arthroscopy.
Pham et al., 2019 [11]	Medication usage, VAS pain score, incidence of adverse events, and patient satisfaction.	A prospective, nonrandomized comparative observational study assigned patients to two different pain regimens following arthroscopic meniscectomy. Regimen 1: Ibuprofen (600 mg every 6-8 hours as needed) and 10 tablets of oxycodone/acetaminophen (5/325 mg as needed for breakthrough pain). Regimen 2: 30-40 tablets of oxycodone/acetaminophen (5/325 mg every 6 hours as needed). There was no significant difference in pain control, satisfaction, and total one-week opioid use between patients prescribed NSAIDs with opioids and those prescribed opioids alone.	Overall, total opioid use did not change in a prospective study where patients were prescribed NSAIDs + opioids or opioids alone. Pain control was also not significantly different between the two groups.
Selley et al., 2021 [16]	iHOT-12 preoperatively, Numeric Pain Rating Scale scores, and opioid consumption postoperatively.	A randomized controlled trial in patients undergoing hip arthroscopy found that prescribing 30 opioid tablets instead of 60 significantly reduced the number of remaining tablets without compromising postoperative pain control. Preoperative factors, including opioid use and iHOT-12, can be used to predict postoperative opioid requirements.	There is no additional benefit in prescribing more opioid tablets postoperatively to manage pain following hip arthroscopy. Evidence demonstrates the importance of assessing a patient’s history to help predict the appropriate postoperative opioid needs.
Lovecchio et al., 2020 [18]	Numeric Rating Scale pain scores and daily opioid consumption.	A randomized controlled trial that analyzed patients who underwent arthroscopic meniscal surgery or ACLR. All meniscus patients and 71% of ACLR patients stopped opioid consumption within one week after surgery. Almost one-third of patients following either surgery (the majority being meniscus surgeries) took no opioids after surgery.	Reasonable prescription quantities are 20 tablets of 5-mg oxycodone after ACLR or 5 tablets of 5-mg oxycodone after arthroscopic meniscus surgery. Orthopedic surgeons should strongly consider patient education regarding expected pain as well as strategies for managing pain.
Barnett et al., 2020 [19]	Pain scores via VAS pain score, opioid consumption, and KOOS score.	Nonrandomized controlled trial was conducted with patients who had undergone ACLR or bridge-enhanced ACLR. Across both groups, oxycodone was oversupplied on average by 46 pills per patient, which correlates to a greater than 70% unused opiate rate. Greater BMI and preoperative pain correlated with greater postoperative opioid use per day. Both groups had similar pain scores from two weeks to two years postoperatively.	Reinforces the evidence that orthopedic surgeons are still overprescribing opioids, which contributes to the increased incidence of opioid dependence.
Johns et al., 2024 [20]	Opioid consumption, a survey regarding continued opioid use, and medication disposal after finishing their prescription.	Randomized controlled trial that followed 176 patients who underwent a variety of different arthroscopic surgeries. Orthopedic surgeons prescribed a mean of 26.6 opioid pills, but patients reported taking an average of 15.5 pills. The MME consumption varied across the different procedures, with a total of 39% of the total opioid prescription being utilized across all five procedures. 7% of patients continued opioid use at three weeks postoperatively, and 24% of the patients with remaining medication intended to keep their medication for use in the future.	This study is consistent with others, highlighting that orthopedic surgeons, in general, overprescribe opioids for arthroscopic surgeries. Overprescribing may lead to patients saving remaining opioids for use in the future, which increases the likelihood of opioid abuse
Degen et al., 2021 [21]	Preoperative and postoperative opioid use via prescription records/fills and use of retrospective data from databases.	The study used retrospective data evaluating 28,830 patients who had undergone hip arthroscopy. The study defined persistent opioid use to be prescriptions continued past 90 days postoperatively; 5.8% of patients fit the criteria. The odds (odds ratio of 5.68) were significantly increased for continuous postoperative use if there was prior preoperative opioid use. Patients with no preoperative use had a 2.5% persistent usage postoperatively compared to the 13.6% who used prior to the operation.	This study emphasizes the need for monitoring and alternative pain management to decrease the risks of persistent opioid usage.
Gil et al., 2019 [22]	Opioid prescription data filled postoperatively.	A retrospective cohort study was conducted, analyzing 59,453 opioid-naive patients who had undergone shoulder arthroscopy procedures. Opioid prescriptions filled between 91-180 days postoperatively were defined as prolonged opioid use, in which 4.3% of patients were identified as prolonged users.	The study showcases potential identified risk factors for new, naive, prolonged opioid usage. It highlights the importance of monitoring opioid usage postoperatively.
Zeng et al., 2013 [23]	VAS pain scores, total opioid consumption in MME, and time to first analgesic request.	A meta-analysis was done on 10 randomized placebo-controlled trials involving 472 patients, analyzing the effects of a single intra-articular morphine injection post-arthroscopic knee procedure. Patients who received the injection had significantly lower pain scores, with an average of -1.1 on VAS pain scores compared to placebo. Patients treated with the intra-articular injection also experienced a longer time to first analgesic request by 77 minutes.	The study highlighted the potential usage of intra-articular morphine as an effective management for postoperative pain. It provides the possibility of using localized analgesia instead of systemic opioid use, minimizing side effects related to systemic use.
Kamdar et al., 2021 [24]	Opioid consumption in OMEs, opioid prescription details, and number of pills consumed via survey/interview.	A retrospective cohort study was done on 112 patients who underwent knee arthroscopy. Patients were prescribed a median of 5 5mg oxycodone pills post-op. The study found that patients consumed considerably fewer pills than prescribed, with a mean count of 1.9 pills (14.3 OMEs). 90% of patients consumed 5 or fewer pills, and 58% didn’t take any. 3% of patients sought refills.	The study suggests that patients consume far less opioids than prescribed. The potential change of prescribing fewer opioids could be a practice to reduce unnecessary exposure to opioids.
Tangtiphaiboontana et al., 2021 [25]	Daily pain diary for the first week after surgery, VAS pain scores, shoulder range of motion, 12-item Short Form Survey, Disabilities of the Arm, and ASES pain scores.	In a randomized, double-blind, placebo-controlled trial of patients undergoing primary arthroscopic rotator cuff surgery, patients were randomized to receive ibuprofen or placebo for two weeks postoperatively in addition to opioid medication. The ibuprofen group consumed significantly less opioids after surgery and had higher mean ASES pain scores and range of flexion at six months.	Using ibuprofen in conjunction with opioids can reduce opioid requirements and facilitate better pain control after arthroscopic shoulder surgery.
Ekelund et al., 2022 [29]	Opioid consumption, pain intensity, time to first opioid rescue, and opioid-free patient proportion.	A randomized, placebo-controlled trial following 107 subjects showed SABER-Bupivacaine reduced pain by 20%, cut opioid use by 66%, and delayed rescue requests by 11 hours in comparison to the placebo group. The utilization of SABER-Bupivacaine also demonstrated no safety concerns.	The trial validates nonopioid analgesics as safe for first-line postoperative arthroscopic pain control. They were shown to be effective, offering equal pain relief with few risks in contrast to opioids.
Kishan et al., 2024 [30]	Opioid consumption (in MMEs), opioid refill requests, office contact, and ASES pain scores.	A randomized controlled trial was conducted on 74 patients placed in three different cohorts: 7-pill, 15-pill, and 23-pill groups. The ASES pain scores in both the 7-pill and 15-pill groups were found to be lower than that of the 23-pill group. However, they found no significant difference in the proportion of patients requesting refills and the number of office contacts with pain-related concerns between the groups.	The study suggests that reducing initial opioid prescriptions for postoperative arthroscopic pain can effectively moderate excess opioid use without compromising pain control. This also supports safer prescribing practices that may help with opioid misuse while maintaining patient recovery.
Buess et al., 2005 [31]	VAS pain scores, SST, and patient satisfaction.	In 96 patients who underwent rotator cuff repairs, there was significant pain reduction in arthroscopic repair in comparison to open repair (p < 0.05). The study also revealed increased functional outcomes through the SST and higher patient satisfaction (92.4%) in contrast to the open repair group (80%).	Not all arthroscopic surgical procedures require opioids postoperatively, which highlights the potential for alternative pain management treatments.
Garvey et al., 2021 [32]	Opioid consumption, VAS pain scores, patient satisfaction, and nonopioid medication use.	Patients who underwent arthroscopic rotator cuff repair were prescribed a multimodal pain regimen of NSAIDs/acetaminophen with as-needed opioids, which showed patients consuming a median of 18 opioid pills over 6.9 days postoperatively. Despite the prescription of opioid pills, 74% of patients achieved satisfactory pain control with limited opioid use, and 94% utilized nonopioid medications and cryotherapy. VAS pain scores demonstrated no association between opioid requirements and tear size, demographics, or preoperative opioid use.	For arthroscopic rotator cuff repair, nonopioids are a safe alternative for postoperative pain control. Utilization of nonopioid multimodal pain regimens reduces opioid exposure without sacrificing outcomes, while also maintaining patient satisfaction.
Jildeh et al., 2022 [34]	VAS pain scores, PROMIS-PI scores, opioid consumption, and adverse events.	Patients undergoing primary arthroscopic rotator cuff repair were randomized to either a multimodal nonopioid pain protocol or standard opioid analgesia. The nonopioid group had significantly lower VAS pain scores on postoperative days one and four, fewer gastrointestinal side effects, and equivalent patient satisfaction compared to the opioid group.	A multimodal nonopioid protocol can be used safely after rotator cuff repair to minimize opioid use without compromising pain control or satisfaction.
Jildeh et al., 2021 [38]	VAS pain scores, PROMIS-PI scores, opioid consumption, and adverse events.	Patients receiving a nonopioid pain protocol had significantly lower VAS and PROMIS-PI scores after controlling for confounders (p < 0.01), with no difference in adverse events or patient satisfaction compared to the opioid group.	A multimodal nonopioid regimen provides effective pain control without increasing adverse events, offering a safe alternative to opioids after arthroscopic shoulder labral surgery.
Elkassabany et al., 2019 [39]	QoR-9 scores, APS-POQ-R pain management domains, and postoperative opioid consumption.	Patients managed with a multimodal perioperative pain protocol had significantly higher QoR-9 scores at 24 and 48 hours, better pain intensity and activity interference scores, and reduced negative emotions compared to the control group. Opioid consumption was significantly reduced for up to three days postoperatively.	Implementing a multimodal perioperative pain protocol after ambulatory shoulder arthroscopy improves early quality of recovery, enhances pain control, and reduces opioid use without compromising patient outcomes.
Jildeh et al., 2021 [40]	VAS pain scores, PROMIS-PI scores, opioid consumption, and adverse events.	Patients undergoing meniscus surgery were randomized to a multimodal nonopioid protocol or standard opioid analgesia. The nonopioid group showed noninferior VAS pain scores, similar PROMIS-PI scores, and no difference in adverse events compared to the opioid group. All patients reported satisfaction with pain control, and no patients required emergency opioid analgesia.	A multimodal nonopioid regimen effectively manages postoperative pain after meniscus surgery while minimizing opioid use, and offering a safe alternative without increasing adverse events or compromising patient satisfaction.

Challenges and limitations

This review must be interpreted in the context of its limitations. This was only a literature search, not a systematic review, and some applicable studies may not have been included due to the search parameters. Only two databases, PubMed and Google Scholar, were screened, which may have limited the breadth of included studies. Different pain scoring systems were used in different studies, making comparisons between studies challenging. Patient populations were not consistent between studies, which makes the application of these findings difficult. The nonopioid pain regimens were also not consistent between studies, and this could be a reason that some achieved statistical significance, while others did not. Sample sizes also varied greatly between studies. Arthroscopy surgery varies regarding what joint is involved, the number of incisions, the pathologies addressed, and patient demographics. This study did not differentiate between different types of arthroscopic surgeries and their indications. Additionally, there were limited studies identifying clear evidence-based protocols for opioid utilization in arthroscopic surgeries. Ultimately, each patient is a unique case, and their personal history needs to be considered when making decisions about postoperative pain control regimens.

Future directions

There are a number of gaps where further research on postoperative pain protocols can be applied. Studies focusing on refining multimodal analgesia to increase their effectiveness can further decrease the number of opioids prescribed post-arthroscopy. Promoting more research in this field can lead to the use of predictive algorithms for personalized pain management approaches based on patient characteristics, including age, comorbidities, mental health history, and SDOH. Considering these factors can minimize opioid overprescription and create a more patient-centered experience. Future investigations with larger sample sizes and uniform pain scales will aid in determining the effectiveness of nonopioid analgesics following arthroscopy. Standardizing data in postoperative pain management research would also help facilitate data comparison across studies while reducing variability. Future investigations should focus on optimizing pain management, reducing opioid misuse, and improving patient experience postoperatively in arthroscopic surgeries.

## Conclusions

Postoperative pain following arthroscopic surgery can often be effectively managed with nonopioid protocols, reducing the risks associated with opioid overprescription. Multimodal analgesia, including NSAIDs, local anesthetics, and intra-articular morphine injections, offers a promising alternative while maintaining comparable pain relief and patient satisfaction. Although opioids may provide faster pain control, nonopioid regimens can minimize side effects such as gastrointestinal discomfort, constipation, nausea, and vomiting. Tailoring pain management to patient history and surgical indications is essential for optimizing outcomes and limiting opioid-related risks.

## References

[1] Treuting R (2000). Minimally invasive orthopedic surgery: arthroscopy. Ochsner J.

[2] Sampognaro G, Harrell R (2023). Multimodal postoperative pain control after orthopaedic surgery. StatPearls.

[3] Moutzouros V, Jildeh TR, Khalil LS, Schwartz K, Hasan L, Matar RN, Okoroha KR (2020). A multimodal protocol to diminish pain following common orthopedic sports procedures: can we eliminate postoperative opioids?. Arthroscopy.

[4] Pathan H, Williams J (2012). Basic opioid pharmacology: an update. Br J Pain.

[5] Queremel Milani DA, Davis DD (2023). Pain management medications. StatPearls.

[6] Gazendam A, Ekhtiari S, Horner NS, Nucci N, Dookie J, Ayeni OR (2021). Perioperative nonopioid analgesia reduces postoperative opioid consumption in knee arthroscopy: a systematic review and meta-analysis. Knee Surg Sports Traumatol Arthrosc.

[7] Thiels CA, Anderson SS, Ubl DS (2017). Wide variation and overprescription of opioids after elective surgery. Ann Surg.

[8] Hartwell MJ, Selley RS, Terry MA, Tjong VK (2020). Can we eliminate opioid medications for postoperative pain control? A prospective, surgeon-blinded, randomized controlled trial in knee arthroscopic surgery. Am J Sports Med.

[9] Gazendam A, Ekhtiari S, Horner NS (2022). Effect of a postoperative multimodal opioid-sparing protocol vs standard opioid prescribing on postoperative opioid consumption after knee or shoulder arthroscopy: a randomized clinical trial. JAMA.

[10] Kamdar PM, Mandava NK, Narula A (2020). Opioid use after knee arthroscopy. Arthrosc Sports Med Rehabil.

[11] Pham H, Pickell M, Yagnatovsky M (2019). The utility of oral nonsteroidal anti-inflammatory drugs compared with standard opioids following arthroscopic meniscectomy: a prospective observational study. Arthroscopy.

[12] Sistani F, Rodriguez de Bittner M, Shaya FT (2023). Social determinants of health, substance use, and drug overdose prevention. J Am Pharm Assoc (2003).

[13] Rogers AH, Bakhshaie J, Orr MF, Ditre JW, Zvolensky MJ (2020). Health literacy, opioid misuse, and pain experience among adults with chronic pain. Pain Med.

[14] Owens PL, Weiss AJ, Barrett ML, Reid LD (2020). Social determinants of health and county population rates of opioid-related inpatient stays and emergency department visits, 2016. Healthcare Cost and Utilization Project (HCUP) Statistical Briefs.

[15] Siddiqui N, Urman RD (2022). Opioid use disorder and racial/ethnic health disparities: prevention and management. Curr Pain Headache Rep.

[16] Selley RS, Hartwell MJ, Alvandi BA, Terry MA, Tjong VK (2021). Risk factors for increased consumption of narcotics after hip arthroscopy: a prospective, randomized control trial. J Am Acad Orthop Surg.

[17] Griffin DR, Parsons N, Mohtadi NG (2012). A short version of the international hip outcome tool (iHOT-12) for use in routine clinical practice. Arthroscopy.

[18] Lovecchio F, Premkumar A, Uppstrom T (2020). Opioid consumption after arthroscopic meniscal procedures and anterior cruciate ligament reconstruction. Orthop J Sports Med.

[19] Barnett S, Murray MM, Liu S (2020). Resolution of pain and predictors of postoperative opioid use after bridge-enhanced anterior cruciate ligament repair and anterior cruciate ligament reconstruction. Arthrosc Sports Med Rehabil.

[20] Johns WL, Johnson EE, Brutico J (2024). Postoperative opioid usage and disposal strategies after arthroscopic procedures in a young cohort: a prospective observational study. Orthop J Sports Med.

[21] Degen RM, McClure JA, Le B, Welk B, Marsh J (2021). Persistent post-operative opioid use following hip arthroscopy is common and is associated with pre-operative opioid use and age. Knee Surg Sports Traumatol Arthrosc.

[22] Gil JA, Gunaseelan V, DeFroda SF, Brummett CM, Bedi A, Waljee JF (2019). Risk of prolonged opioid use among opioid-naïve patients after common shoulder arthroscopy procedures. Am J Sports Med.

[23] Zeng C, Gao SG, Cheng L (2013). Single-dose intra-articular morphine after arthroscopic knee surgery: a meta-analysis of randomized placebo-controlled studies. Arthroscopy.

[24] Kamdar PM, Liddy N, Antonacci C (2021). Opioid consumption after knee arthroscopy. Arthroscopy.

[25] Tangtiphaiboontana J, Figoni AM, Luke A, Zhang AL, Feeley BT, Ma CB (2021). The effects of nonsteroidal anti-inflammatory medications after rotator cuff surgery: a randomized, double-blind, placebo-controlled trial. J Shoulder Elbow Surg.

[26] Gazendam A, Ekhtiari S, Horner NS (2021). Protocol for a multicenter randomized controlled trial comparing a non-opioid prescription to the standard of care for pain control following arthroscopic knee and shoulder surgery. BMC Musculoskelet Disord.

[27] O Cathasaigh M, Read MR, Atilla A, Schiller T, Kwong GP (2018). Blood concentration of bupivacaine and duration of sensory and motor block following ultrasound-guided femoral and sciatic nerve blocks in dogs. PLoS One.

[28] Shafiei FT, McAllister RK, Lopez J (2023). Bupivacaine. StatPearls.

[29] Ekelund A, Peredistijs A, Grohs J, Meisner J, Verity N, Rasmussen S (2022). SABER-bupivacaine reduces postoperative pain and opioid consumption after arthroscopic subacromial decompression: a randomized, placebo-controlled trial. J Am Acad Orthop Surg Glob Res Rev.

[30] Kishan A, Pearson ZC, Li SS, Pressman Z, Ahiarakwe U, Pathiravasan CH, Srikumaran U (2024). How low can we go? A randomized controlled trial of low-quantity initial opioid prescriptions for shoulder surgery. J Shoulder Elbow Surg.

[31] Buess E, Steuber KU, Waibl B (2005). Open versus arthroscopic rotator cuff repair: a comparative view of 96 cases. Arthroscopy.

[32] Garvey KD, Lowenstein NA, Piana LE, Arant KR, Chang Y, Matzkin EG (2021). Satisfactory pain management with minimal opioid use after arthroscopic rotator cuff repair. Arthrosc Sports Med Rehabil.

[33] Stephan BC, Parsa FD (2016). Avoiding opioids and their harmful side effects in the postoperative patient: exogenous opioids, endogenous endorphins, wellness, mood, and their relation to postoperative pain. Hawaii J Med Public Health.

[34] Jildeh TR, Abbas MJ, Hasan L, Moutzouros V, Okoroha KR (2022). Multimodal nonopioid pain protocol provides better or equivalent pain control compared to opioid analgesia following arthroscopic rotator cuff surgery: a prospective randomized controlled trial. Arthroscopy.

[35] Hörl WH (2010). Nonsteroidal anti-inflammatory drugs and the kidney. Pharmaceuticals (Basel).

[36] Leth MF, Bukhari S, Laursen CC (2022). Risk of serious adverse events associated with non-steroidal anti-inflammatory drugs in orthopaedic surgery. A protocol for a systematic review. Acta Anaesthesiol Scand.

[37] Brummett CM, Waljee JF, Goesling J (2017). New persistent opioid use after minor and major surgical procedures in US adults. JAMA Surg.

[38] Jildeh TR, Khalil LS, Abbas MJ, Moutzouros V, Okoroha KR (2021). Multimodal nonopioid pain protocol provides equivalent pain control versus opioids following arthroscopic shoulder labral surgery: a prospective randomized controlled trial. J Shoulder Elbow Surg.

[39] Elkassabany NM, Wang A, Ochroch J, Mattera M, Liu J, Kuntz A (2019). Improved quality of recovery from ambulatory shoulder surgery after implementation of a multimodal perioperative pain management protocol. Pain Med.

[40] Jildeh TR, Okoroha KR, Kuhlmann N, Cross A, Abbas MJ, Moutzouros V (2021). Multimodal nonopioid pain protocol provides equivalent pain versus opioid control following meniscus surgery: a prospective randomized controlled trial. Arthroscopy.

